# Maternal transcription of non-protein coding RNAs from the PWS-critical region rescues growth retardation in mice

**DOI:** 10.1038/srep20398

**Published:** 2016-02-05

**Authors:** Timofey S. Rozhdestvensky, Thomas Robeck, Chenna R. Galiveti, Carsten A. Raabe, Birte Seeger, Anna Wolters, Leonid V. Gubar, Jürgen Brosius, Boris V. Skryabin

**Affiliations:** 1 Institute of Experimental Pathology (ZMBE), University of Muenster, Von-Esmarch-Str. 56, D-48149 Münster, Germany; 2 Department of Medicine (TRAM), University Hospital of Muenster, Von-Esmarch-Str. 56, D-48149 Münster, Germany; 3 Institute of Evolutionary and Medical Genomics, Brandenburg Medical School (MHB), D-16816 Neuruppin, Germany

## Abstract

Prader-Willi syndrome (PWS) is a neurogenetic disorder caused by loss of paternally expressed genes on chromosome 15q11-q13. The PWS-critical region (*PWScr*) contains an array of non-protein coding IPW-A exons hosting intronic *SNORD116* snoRNA genes. Deletion of *PWScr* is associated with PWS in humans and growth retardation in mice exhibiting ~15% postnatal lethality in C57BL/6 background. Here we analysed a knock-in mouse containing a 5′HPRT-LoxP-Neo^R^ cassette (5′LoxP) inserted upstream of the *PWScr*. When the insertion was inherited maternally in a paternal *PWScr*-deletion mouse model (*PWScr*^*p*−/*m5*′*LoxP*^), we observed compensation of growth retardation and postnatal lethality. Genomic methylation pattern and expression of protein-coding genes remained unaltered at the PWS-locus of *PWScr*^*p*−/*m5*′*LoxP*^ mice. Interestingly, ubiquitous Snord116 and IPW-A exon transcription from the originally silent maternal chromosome was detected. *In situ* hybridization indicated that *PWScr*^*p*−/*m5*′*LoxP*^ mice expressed Snord116 in brain areas similar to wild type animals. Our results suggest that the lack of PWScr RNA expression in certain brain areas could be a primary cause of the growth retardation phenotype in mice. We propose that activation of disease-associated genes on imprinted regions could lead to general therapeutic strategies in associated diseases.

The human SNURF-SNRPN domain located at 15q11q13 (murine 7qC) is subject to genomic imprinting controlled by a bipartite imprinting centre (IC)^1^ (Fig. 1). This region spans 3–4 Mbp, and is considered to be the largest human imprinting cluster identified thus far. Imprinting defects or chromosomal rearrangements/deletions within this locus result in the development of two clinically distinct neurogenetic disorders – the Prader-Willi (PWS) and Angelman (AS) syndromes, respectively.

AS (MIM 105830) is a severe neurogenetic disorder that is associated with mental retardation, epilepsy, movement disorder and abnormal behaviour. AS is caused by the deletion or inactivating mutations of the UBE3A gene that encodes E3 ubiquitin ligase. In human neurons, UBE3A is exclusively expressed from the maternal chromosome.

PWS (MIM 176270) is a complex neurogenetic disorder with a prevalence of 1 in 10,000 to 1 in 30,000 individuals. Typically, progression of PWS can be subdivided into two main stages: (i) early – characterized by muscular hypotonia resulting in failure to thrive and (ii) late - mostly defined by severe obesity. Additional symptoms common to PWS patients include short stature, small hands and feet, obsessive-compulsive behaviour and mild intellectual disability (for review^2^,^3^).

PWS patients lack the paternal expression of imprinted genes located within the PWS locus. Approximately 70% of PWS patients harbour paternally inherited deletions of the entire imprinting domain, and approximately 25% of all cases are due to maternal uniparental disomy (two copies of maternal chromosomes)^3^,^4^. At most, 3% of PWS patients display genomic imprinting defects^3^,^4^.

Several paternally expressed protein-coding genes map to this locus, including *NECDIN (NDN)*, *MAGEL2*, *MKRN3*, *C15orf2* (recently designated as *NPAP1*), and the bi-cistronic *SNURF-SNRPN* (Fig. 1A). Apart from protein-coding genes, this locus also encodes various non-protein-coding RNA (npcRNA) genes; for instance the poorly characterized U-exons and PWRN1/PWRN2 long non-protein-coding transcripts localized between *NDN* and *SNURF-SNRPN* genes. In addition, the domain harbours the long U-*UBE3A-ATS* npcRNA transcript, which spans approximately ~450 kb. It initiates from the U exons upstream of the *SNURF/SNRPN* gene and extends to the *UBE3A* gene in antisense orientation (Fig. 1A,B)^5^. The PWS locus also encodes numerous paternally expressed C/D box snoRNAs. Most of them, if not all are processed from introns of the U-*UBE3A-ATS* transcript^5^. This arrangement includes two large tandemly repeated clusters of *SNORD116* (*HBII-85 snoRNA)* and *SNORD115 (HBII-52 snoRNA)* RNA genes containing 29 and 47 copies, respectively^6^. Additional snoRNA genes, *SNORD107* (*HBII-436*), *SNORD64* (*HBII-13*), and *SNORD108* (*HBII-437*) are encoded by single and *SNORD109A/B* (*HBII-438a/438b*) by two copies^5^,^6^ (Fig. 1A). In mouse, the PWS/AS locus maps to chromosome 7C (Fig. 1B). The gene order of the PWS/AS locus and mono-allelic expression in brain are largely conserved. However, in mouse the *NPAP1*, *SNORD108* and *SNORD109A/B* orthologs are missing, whereas the human locus is devoid of the *Frat3* (*Peg12*) gene^6^,^7^ (Fig. 1B).

Analysis of different PWS-locus deletions in mouse models and gene expression in patients with chromosomal translocations predicted the *Snord116* gene array to define the PWS critical region (*PWScr*)^8^,^9^,^10^,^11^. Previously, we exclusively deleted the *Snord116* gene cluster via “chromosome engineering” in mice^12^. Upon maternal transmission of the deleted allele (*PWScr*^*p*+*/m*−^), no phenotypic abnormalities were detected. However, if mice were carrying the paternal deletion of the *Snord116* cluster (*PWScr*^*p*−/*m*+^), postnatal growth retardation was observed^12^. Furthermore, ~15% postnatal lethality was detected in C57BL/6 genetic background, but not in FVB/N or BALB/c^12^. An independent study eliminating a similar overlapping region in mice, ~45 kb larger than that reported by Skryabin *et al.* (2007), provided similar results^13^. In addition, deficiencies in motor learning, increased anxiety, hyperphagia, and altered metabolism were reported^13^. No adolescent obesity was observed in either mouse model^12^,^13^. Subsequently, several PWS patients were identified that carry a microdeletion of the *SNORD116* gene cluster, strongly supporting that loss of the paternal *SNORD116* cluster is responsible for key characteristics of the PWS phenotype^14^,^15^,^16^,^17^.

In the present study, we analysed a knock-in (KI) mouse model that harbours a 5′HPRT-LoxP-Neo^R^ (hypoxanthine-guanine phosphoribosyltransferase, LoxP and neomycin resistance gene) (5′LoxP) containing cassette ∼27 kb upstream from the *Snord116* gene array. We provide biochemical evidence that the insertion does not alter a methylation pattern within the PWS imprinting centre (PWS-IC), and also leaves the expression of protein-coding genes largely unaltered. By contrast, transcription of both the *Snord116* and *Snord115* gene clusters from the maternal chromosome was observed. We demonstrated that the maternal expression of the Snord116 gene cluster in the *PWScr*^*p*−/*m5*′*LoxP*^ mouse model rescued both postnatal lethality and growth retardation, which is specifically associated with the *PWScr*^*p*−/*m*+^ genetic background. Our data emphasized the importance of non-protein-coding RNAs in the etiology of PWS.

## Results

### Generation of *PWScr* ^*p*−/*m5*′*LoxP*^ mice and growth detection

**Figure 1:**
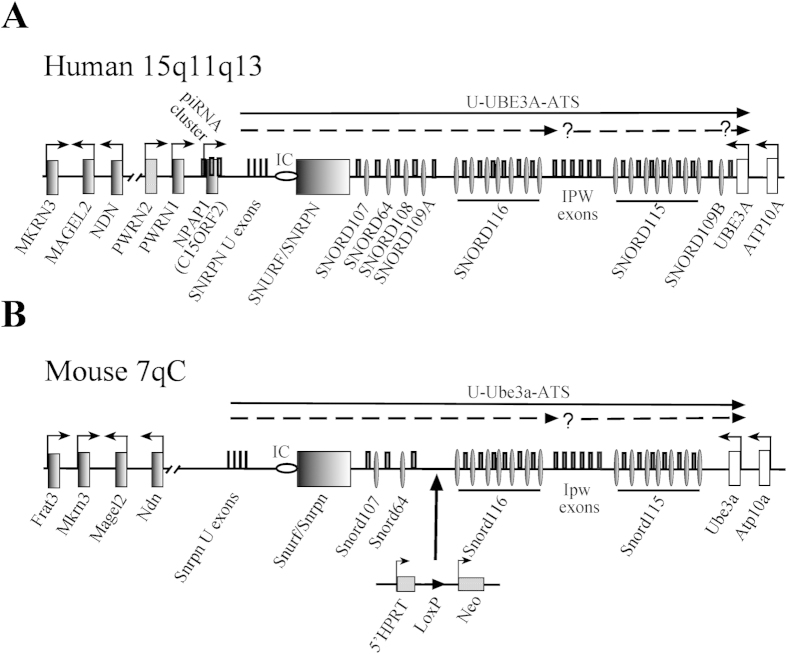
Schematic representation of the PWS-locus in human and mice. (**A**) Human chromosome 15q11-q13 region (not drawn to scale). Rectangles or thin ovals denote protein coding gene or snoRNA gene locations and the imprinting center (IC) is shown by a horizontal oval; thin rectangles above the midline denote non-protein coding exons. Arrows indicate promoters and the direction of transcription. The two broken arrows under the top arrow showing the U-UBE3A antisense transcript harboring the two SNORD116 and SNORD115 clusters indicate putative additional primary transcripts with a possible additional promoter upstream from the SNORD115 cluster. (**B**) Schematic representation of the mouse PWS-locus on chromosome 7; the 5′HPRT-LoxP-Neo^R^ targeting cassette is indicated (not drawn to scale).

For the construction of the *PWScr*^*p*−/*m5*′*LoxP*^ KI mouse model, hypoxanthine-guanine phosphoribosyltransferase (*HPRT*)-deficient AB2.2 ES cells were modified by homologous recombination (HR) using the targeting construct 5′*HPRT*/*PWScr*_targ as the first step in generating the previously reported *PWScr*^*p*−/*m*+^ mice (Supplementary Fig. 1)^12^. The construct contained 5′-domains of the *HPRT* gene controlled by the *Pgk* promoter, the LoxP site and the neomycin phosphotransferase (*Neo*) gene, which is transcribed from the promoter of the gene encoding the large subunit of RNA polymerase II (RNAP II) (Fig. 1B, Supplementary Fig. 1C).

The 5′ cassette containing the LoxP site was placed upstream of the *Snord116* gene cluster (Fig. 1B Supplementary Fig. 1C).

We obtained a single (from ~250) positive ES clone, which harboured the targeting cassette in the desired location. Expanded ES cells were injected into blastocysts. Chimeras with germ-line transmission were identified by Southern blot analysis as described previously^12^. Pups of chimeric mice that contained the corresponding 5′LoxP cassette inserted at the desired position, (downstream from *Snord64* and upstream from the *Snord116* gene cluster) did not reveal discernible phenotypic differences compared to wild type siblings. The heterozygous *PWScr*^*p5*′*LoxP/m*+^ pups were intercrossed to establish a homozygous KI mouse line. Next, we generated heterozygous KI female mice with modified maternal chromosome (*PWScr*^*p*+/*m5*′*LoxP*^) and crossed them with *PWScr*^*p*−/*m*+^ males to generate *PWScr*^*p*−/*m5*′*LoxP*^, *PWScr*^*p*−/*m*+^ and wild type siblings.

*PWScr*^*p*−/*m5*′*LoxP*^ pups were slightly, yet distinguishably smaller than their wild-type counterparts when weighed on postnatal day 12 (P12). However, the growth differences became imperceptible after three weeks (P21).

**Figure 2:**
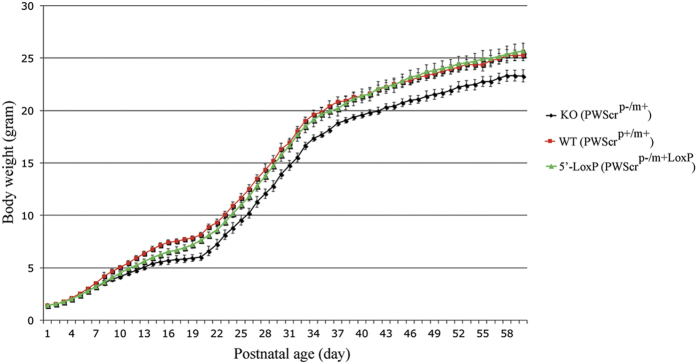
Growth dynamics of *PWScr*^*p*−/*m5*′*LoxP*^, *PWScr*^*p*−/*m*+^ and male wild type mice. Curves show the growth dynamics of 86 male mice. The red line shows the weight gain of 30 wild type mice; the green line corresponds to 30 *PWScr*^*p*−/*m5*′*LoxP*^ mice and the black line corresponds to 26 *PWScr*^*p*−/*m*+^ mice. Bars indicate standard deviation. Weight mean, observed standard deviation, and confidence interval for each time point of the investigated mice, are calculated with a confidence level of 95% (p = 0.05) in Supplementary Table 1.

We monitored weight increase of individual *PWScr*^*p*−/*m5*′*LoxP*^ mice over several weeks in comparison to sibling *Snord116* knockout mice (*PWScr*^*p*−/*m*+^) and wild type (*PWScr*^*p*+*/m*+^) mice respectively (Fig. 2). The overall weight gain of n = 30 males and n = 26 females of the *PWScr116*^*p*−/*m5*′*LoxP*^ genotype were compared to n = 30 males and n = 30 females of wild type and n = 26 males and n = 28 females of *PWScr*^*p*−/*m*+^ animals (Fig. 2; Supplementary Fig. 2; Supplementary Table 1). Analysis of *PWScr*^*p*−/*m5*′*LoxP*^ growth dynamics revealed a mild growth delay between postnatal day 7 and 19. However, with increasing age the difference vanished and became mostly insignificant in *PWScr*^*p*−/*m5*′*LoxP*^ P21 males and P22 females compared to wild type mice of identical age (Fig. 2, Supplementary Fig. 2), and no increase in postnatal lethality was detected for *PWScr*^*p*−/*m5*′*LoxP*^ mice (Supplementary Fig. 3). Hence, measurements and analysis of body weight alteration of *PWScr*^*p*−/*m5*′*LoxP*^, *PWScr*^*p*−/*m*+^ and wild type mice indicated that after the first 3 weeks there was a complete rescue of the growth retardation phenotype in mice harbouring the maternal 5′LoxP cassette compared to *PWScr*^*p*−/*m*+^ mice with an unmodified maternal chromosome (Fig. 2; Supplementary Table 1). Consistent with our previous results, postnatal growth retardation of *PWScr*^*p*−/*m*+^ mice was observed beginning on postnatal day 5 in males and on day 6 in females lasting into adulthood (Fig. 2)^12^.

### Expression of genes from PWS/AS locus in mouse models

**Figure 3:**
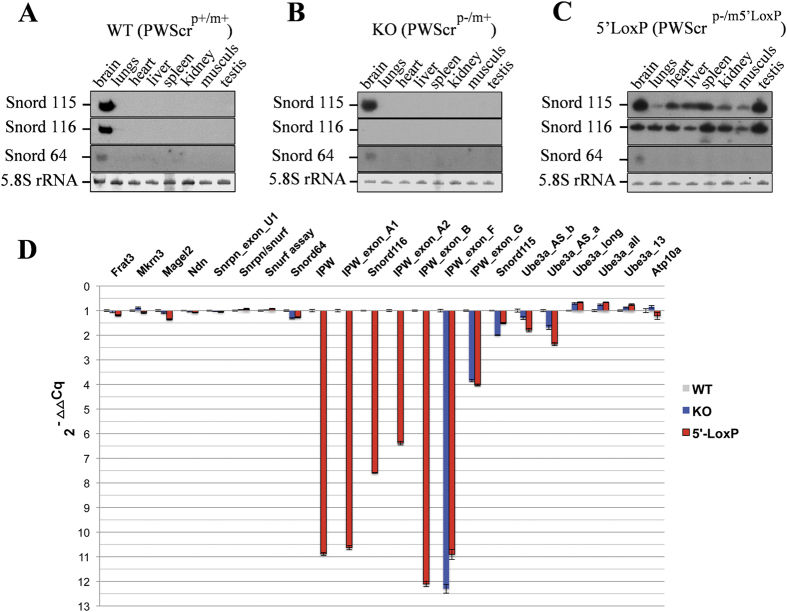
Expression of PWS/AS locus genes in *PWScr*^*p*−/*m5*′*LoxP*^, *PWScr*^*p*−/*m*+^ and wild type mice. (**A**–**C**) Northern blot analyses of PWS-locus snoRNAs from different tissues of wild type, *PWScr*^*p*−/*m*+^ and *PWScr*^*p*−/*m5*′*LoxP*^ mice. Ethidium bromide-stained 5.8 S rRNA is shown as RNA loading control. Tissues and mouse genotypes are indicated on the top of each blot panel. (**D**) RT-qPCR analysis of PWS/AS-locus genes; the fold change is represented as 2^−ΔΔCq^. Blue and red bars represent the RNAs expression fold change values of *PWScr*^*p*−/*m*+^ and *PWScr*^*p*−/*m5*′*LoxP*^ mice, respectively. The plot represents values of Supplementary Table 2.

To delineate the effects of the maternally inherited 5′LoxP cassette at the molecular level, we analysed the expression of non-protein-coding and protein-coding genes within the PWS/AS locus. As mentioned, the mouse PWS locus encodes the *Snord64* gene and two large paternally expressed snoRNA clusters: *Snord116* and *Snord115*, respectively. In contrast to human, the expression of the latter two snoRNAs is restricted to the mouse brain^1^,^6^,^18^,^19^ (Fig. 3).

We investigated the effects of the 5′LoxP cassette on expression patterns of the distal *Snord116* and *Snord115* gene clusters. When total RNA from different tissues isolated from 3 male and 3 female mice of wild type, *PWScr*^*p*−/*m5*′*LoxP*^ and *PWScr*^*p*−/*m*+^ genotypes were analysed by Northern blot hybridization, we observed ubiquitous RNA expression of both snoRNAs in *PWScr*^*p*−/*m5*′*LoxP*^ mice (Fig. 3C). In contrast, we did not observe any change in the brain specific expression pattern of Snord64 RNA located upstream of the inserted cassette (Figs 1 and 3A–C). Since expression of *Snord115* in *PWScr*^*p*−/*m*+^ and both snoRNA clusters in wild type mice is restricted to brain (Fig. 3A,B), we conclude that the inserted 5′LoxP cassette results in ubiquitous transcriptional activation of the maternal allele. Brain-specific expression of Snord64 RNA remained unaltered and suggested that only the snoRNA genes located downstream from the transgene insertion were affected (Fig. 3A–C). Interestingly, the observed activation of transcription from the maternal chromosome in *PWScr*^*p*−/*m5*′*LoxP*^ mice suggests that the transcriptional activation of *Snord116* and *Snord115* gene clusters is not controlled by the PWS-IC centre and is not maternally silenced.

Next, we performed a comprehensive expression analysis of protein-coding and non-protein-coding genes within the PWS/AS locus using reverse transcription quantitative real-time PCR (RT-qPCR)^19^,^20^. Total brain RNA from three male animals of each genotype (wild type, *PWScr*^*p*−/*m5*′*LoxP*^ and *PWScr*^*p*−/*m*+^) at postnatal day 7 was extracted independently and RT-qPCR analysis was performed in triplicate for each sample (Fig. 3D, Supplementary Table 2). The mouse PWS – locus harbours several paternally expressed protein-coding genes, including *Frat3*, *Mkrn3*, *Magel2*, *Ndn* (Necdin) and bi-cistronic *Snurf*/*Snrpn* (Fig. 1). Their expression is tightly controlled by the PWS-IC that maps to a ~6 kb region between −3.7 kb and +2.3 kb relative to exon 1 of the mouse *Snrpn* gene^21^. Complete or partial deletion of this region results in complete or at least significant loss of gene expression in the PWS locus^21^,^22^,^23^. The RT-qPCR analysis did not reveal significant changes in the corresponding expression levels of all investigated mRNAs within the *PWScr*^*p*−/*m5*′*LoxP*^ genotypes when compared to both to wild type and *PWScr*^*p*−/*m*+^ animals, respectively (Fig. 3D, Supplementary Table 2).

Next, we examined the expression profile of PWS-locus encoded non-protein-coding RNAs. Most of them, if not all, are presumed to be processed from the long U-Ube3A-ATS RNA transcript, which initiates from a U-exon on the paternal un-methylated chromosomal region(s), located upstream from the PWS-IC centre (Fig. 1)^24^. Analogous to the PWS locus protein coding genes, the IC tightly controls expression of the U-Ube3A-ATS pre-RNA and consequently the snoRNAs^1^,^21^. In mouse, the transcription of U-Ube3A-ATS pre-RNA is restricted to the neurons of most areas of the brain^3^. The RNA contains various alternatively spliced exons, which generate numerous large RNAs (identified as ESTs – expressed sequence tags in databases) and snoRNAs. Among the ESTs, there are U-exons, Ipw, Ipw-B – F, repetitive subtypes of Ipw-A and Ipw-G exons, Ube3a *cis*-antisense transcripts etc. (Fig. 1; Supplementary Fig. 1)^6^. Imprinted IPW exons A and G flank the Snord116 and Snord115 RNA copies, respectively (Fig. 1)^6^. To evaluate expression of different PWS locus non-protein-coding transcripts, we have designed primers targeting different U-Ube3a-ATS RNAs and performed RT-qPCR (Supplementary Table 3). Overall, we did not observe any significant differences in the expression of npcRNAs that are located upstream from the 5′LoxP cassette insertion in the *PWScr*^*p*−/*m5*′*LoxP*^ mice compared to wild type and *PWScr*^*p*−/*m*+^ animals (Fig. 3D; Supplementary Table 2). The results correlate well with unaltered expression of paternally imprinted protein-coding genes and suggest that regulation by the PWS-IC-centre is not affected (Fig. 3D).

As expected, RT-qPCR analysis of RNA samples extracted from the brains of *PWScr*^*p*−/*m*+^ mice did not reveal expression of Ipw, IpwA1/A2, Snord116 and IpwB transcripts. In contrast, those RNAs were detected in *PWScr*^*p*−/*m5*′*LoxP*^ brain samples, once more indicative of maternal gene activation in the KI mice. However, the maternally inherited 5′LoxP cassette led to significantly lower expression values of the aforementioned RNAs when compared to wild type mice (Fig. 3D, Supplementary Table 2). We observed a 7.5 and ~12 fold decrease in the expression levels of Snord116 RNA and IPW transcripts, respectively (Fig. 3D, Supplementary Table 2). In line with our previous observation in *PWScr*^*p*−/*m*+^ mouse models, the expression of IPW-G exons in *PWScr*^*p*−/*m5*′*LoxP*^ was also reduced (∼4-fold) in comparison to wild type animals, but the expression levels of Snord115 as detected by RT-qPCR were only slightly lower (∼1.5-fold)^12^. This could be due to different stabilities of the RNAs. Notably, the expression level of Snord115 in *PWScr*^*p*−/*m*+^ mice was almost 2-fold lower than in wild type animals, which was not detected in our previous study using Northern blot analysis^12^. Interestingly, we also observed a slight decrease of Ube3a antisense transcripts that potentially led to a small increase of Ube3a mRNA isoforms expression in *PWScr*^*p*−/*m*+^ and *PWScr*^*p*−/*m5*′*LoxP*^ mouse models (Fig. 3D, Supplementary Table 2).

Although we could detect transcription from both RNA polymerase II promoters of the 5′HPRT-LoxP-NeoR cassette, we were not able to conclusively determine the promoter(s) driving expression of the primary transcript harboring the IPW exons and snoRNAs on the maternal chromosome. In any event, our results demonstrate that even a lower expression level of maternal *PWScr* locus transcription was sufficient to compensate for the growth retardation phenotype associated with the *PWScr*^*p*−/*m*+^ mice^12^. Yet, the lower expression level might be the cause of moderate growth retardation observed during the first three weeks of postnatal development.

### Maternal inheritance of the 5′LoxP cassette does not affect methylation of the PWS-IC

**Figure 4:**
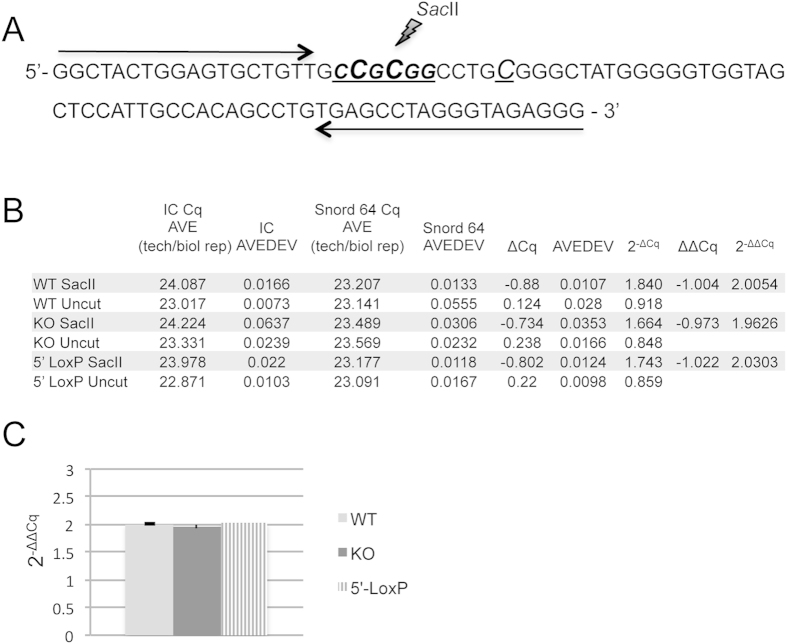
PWS-IC-center CpG methylation analysis. (**A**) The mouse PWS-IC genomic region selected for the qPCR assay; cytosine residues that are methylated on the maternal chromosome are shown as capitalized, underlined and italic letters. The *Sac*II endonuclease recognition site is CCGCGG. Quantitative PCR primers are indicated by a black arrows. (**B**) A summary of the qPCR data is detailed in Supplementary Table 4. For each mouse genotype (WT: wild type; KO: *PWScr*^*p*−/*m*+^ and 5′ LoxP: *PWScr*^*p*−/*m5*′*LoxP*^), six brains were used to isolate DNA samples. Each sample was analysed in triplicate. DNA sample*s either* digested and untreated *are indicated by Sac*II and Uncut respectively. Columns: IC Cq AVE (tech/biol rep) is the average of Cq values obtained from technical and biological replicates per category during the qPCR of the PWS-IC region; IC AVEDEV is the average of standard deviations obtained from all replicates per category during qPCR of the PWS-IC region; Snord64 Cq AVE (tech/biol rep): the average of Cq values obtained during qPCR of the Snord64 gene region; Snord64 AVEDEV: is the respective average standard deviation. (**C**) Chart visualising 2^−ΔΔCq^ values obtained by qPCR show no differences in CpG methylation of the PWS-IC region among the different mouse genotypes.

The imprinted gene expression within the PWS-locus is tightly controlled by the PWS-IC, which is differentially methylated during development. CpG DNA methylation is restricted to the maternal chromosome and causes gene silencing in the locus; the paternal allele, however, remains unmodified and hence transcriptionally active. In mice, the methylation of the PWS-IC is established in oocytes and maintained throughout embryonic development into adulthood^1^,^3^. Loss of maternal IC methylation results in the activation of PWS gene expression from the maternal chromosome^23^. To address possible changes of altered methylation patterns of the PWS-IC in *PWScr*^*p*−/*m5*′*LoxP*^ mice compared to wild type and *PWScr*^*p*−/*m*+^ animals, we conducted quantitative real-time PCR (qPCR) analysis. The *Sac*II methylation sensitive restriction endonuclease site in the PWS-IC genomic CpG region was chosen (Fig. 4A). DNA samples from six mice (3 per gender) for each genotype were analysed. Since each round of PCR amplification results in roughly a 2-fold increase in the amount of product, the detected 2-fold differences between *Sac*II digested and intact DNA samples from *PWScr*^*p*−/*m5*′*LoxP*^, wild type, and *PWScr*^*p*−/*m*+^ mice indicates that ~50% of the template was cleaved in the endonuclease digested samples (Fig. 4B,C; Supplementary Table 4). Hence, qPCR analysis revealed that CpG methylation of the PWS-IC *Sac*II site on the maternal chromosome was not affected by inheritance of the 5′HPRT-LoxP-NeoR cassette insertion (Fig. 4).

### Maternal expression of Snord116 in areas of the brain

**Figure 5:**
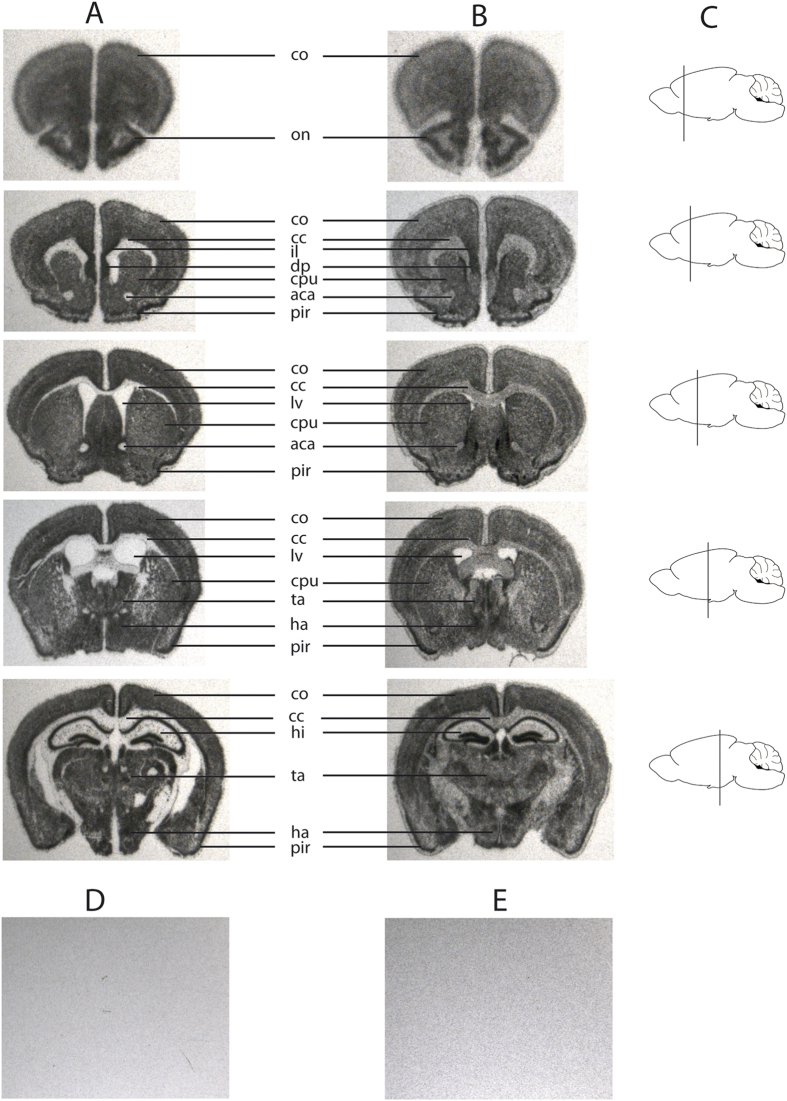
Snord116 *in situ* hybridization (ISH) of wild type and *PWScr*^*p*−/*m*+*5*′*LoxP*^ mouse sagittal brain sections (**A**) Brain sections of wild type mice, the exposure time was one day (**B**) brain sections of *PWScr*^*p*−/*m*+*5*′*LoxP*^ mice. ISH is performed with Snord116 antisense probe. (**A**,**B**) Mouse brain arias are denoted as follows: cc: corpus callosum; co: cortex; ha: hypothalamic area; hi: hippocampus; lv: lateral ventricle; on: olfactory nucleus; pu: putamen; ta: thalamic area; pir: piriformal cortex; il: infralimbic cortex; dp: dorsal peduncular cortex. Due to the lower expression levels of Snord116 in the KI mouse, the exposure time had to be increased from 1 to 7 days. (**C**) Schematic representation of the cuts in sagittal brain sections. (**D**) Negative control, example of brain sections of *PWScr*^*p*−/*m*+^ mice hybridized with Snord116 antisense probe. (**E**) Example of brain sections of wild type mice hybridized with Snord116 sense probe. (**D**,**E**) The exposure time was 4 days.

Since, insertion of the 5′LoxP cassette altered the transcriptional control of *PWScr*, we investigated the expression of Snord116 in different brain regions of *PWScr*^*p*−/*m5*′*LoxP*^ mice in comparison to wild type animals. *In situ* hybridization (ISH) with a Snord116 antisense probe was performed on floating brain sections obtained from six to eight week old mice. The Snord116 distribution between wild type and *PWScr*^*p*−/*m5*′*LoxP*^ mice was similar (Fig. 5A–C). As negative control, brain sections isolated from *PWScr*^*p*−/*m*+^ were used. In addition, ISH was performed with a control Snord116 sense probe. No signals were observed in control experiments, even after long exposures (Fig. 5D,E). The strongest ISH signals in brains of both Snord116-expressing mouse lines (wild type and *PWScr*^*p*−/*m5*′*LoxP*^) were observed in the hypothalamus, thalamus, hippocampus, anterior olfactory nucleus, piriformal cortex, infralimbic cortex, putamen and dorsal peduncular cortex (Fig. 5). Other regions exhibited moderate Snord116 expression signals. Differences in snoRNA116 expression between wild type and *PWScr*^*p*−/*m5*′*LoxP*^ mice were noted; for example, in corpus callosum and anterior commissure areas of the brain. The corpus callosum and anterior commissure are bundles of nerve fibers (white matter tracts) involved in interhemispheric communication. They are composed mainly of axons and glial cells. In wild type mice, expression of Snord116 RNA is restricted to neurons, where it is predominantly localised in the nucleolus (with low levels in nucleoplasm)^18^. In contrast, robust expression of Snord116 in *PWScr*^*p*−/*m5*′*LoxP*^ mice was observed in non-brain tissue (Fig. 3A), and ISH signals in the corpus callosum and anterior commissure areas of KI mouse brain suggest expression of snoRNA in glial cells (Fig. 5). Nevertheless, although Snord116 was detected in the glial cells of *PWScr*^*p*−/*m5*′*LoxP*^ mice, the overall brain area patterns of neuronal snoRNA expression between KI and wild type mice was quite similar (Fig. 5, Supplementary Fig. 5).

## Discussion

Studies of different deletion mouse models and PWS patients have identified a *PWS*-critical region. This region contains the *SNORD116* gene copies flanked by repeated IPW-A exons (elsewhere termed IPW116 or host gene exons – 116HG)^12^,^13^,^14^,^15^,^16^,^17^. Thus far, an important question still remains unanswered. Is the deletion of unknown regulatory elements or lack of non-protein coding RNAs causative of PWS in patients? Recent studies on the activation of PWS-locus gene expression from the maternal chromosome in mice and in PWS-specific induced pluripotent stem cells (iPSCs) indicated the importance of RNAs derived from this region^23^,^25^. However, expression of all PWS-locus protein coding and non-protein coding genes was observed and the actual contribution of the Snord116 genes cluster could not be dissected^23^. Here, based on a KI mouse model we could demonstrate that activating the maternal chromosome region encompassing Snord116 results in rescue of growth retardation and postnatal lethality. The expression levels of Snord116 and IPW-A non-protein coding exons observed in *PWScr*^*p*−/*m5*′*LoxP*^ mice were considerably lower than those in wild type animals. Although mild growth retardation was observed during the early postnatal period, growth differences between *PWScr*^*p*−/*m5*′*LoxP*^ and wild type littermates were not discernible after postnatal day 21 and 22 in males and females, respectively. Therefore, our data suggest that expression and to some extent quantity of non-protein coding RNAs play an important role during early postnatal development in mice.

Recently, transcription of the IPW exon containing RNAs had been linked to regulation of maternally expressed genes (MEGs) in the human DLK1-DIO3 imprinted locus in an iPSCs model of PWS. This study suggests that RNA interaction with histone methyltransferase G9A targets the imprinted DNA methylation region (iDMR) in the DLK1-DIO3 locus *in trans*^26^. However, the IPW exons and many iDMRs do not show sequence conservation between mammals in general, and human and mouse in particular^6^,^27^. Therefore, the involvement of IPW-A exons containing long RNA in the regulation of MEGs from Dlk1-Dio3 imprinted domain in mice remains unclear and will be subject to further investigation. Recently, mouse IPW-A exon containing RNA was suggested to regulate diurnal energy expenditure^28^.

In the PWS critical region, the *Snord116* genes exhibit the largest degree of sequence similarity between various mammalian species. Consequently, the focus of investigation was directed to potential snoRNA function. In order to discriminate whether the snoRNA or the long host transcript is responsible for the phenotype, compensation had been attempted by generating *Snord116* transgenic mouse lines embedded in introns of different host genes^13^. A single copy *Snord116* gene, inserted within nucleolin intron 11, revealed, as expected, relatively low level of expression^13^. The mouse lines failed to rescue growth retardation and lethality of the *Snrpn* to *Ube3A* deletion mouse model^13^,^29^. It is difficult to make any conclusions, as the exact brain localization of the transgene-derived Snord116 was not reported. Our results demonstrate that if Snord116 is responsible for the growth retardation phenotype observed in *PWScr*^*p*−/*m*+^ mice then ~7.5 fold decrease in Snord116 RNA expression is sufficient to rescue the growth retardation phenotype after postnatal day 21 and 22 in males and females, respectively (Figs 2 and 3D and Supplementary Fig. 2). Importantly, the *in situ* hybridization experiments revealed that activation of Snord116 expression from the maternal chromosome in *PWScr*^*p*−/*m5*′*LoxP*^ mice overlaps with the brain areas of wild type mice that express this snoRNA from the paternal chromosome (Fig. 5).

We have preliminary results pertaining to a transgenic mouse line expressing two copies of mouse and one of rat Snord116 processed from introns of a different host gene, namely EGFP (*TgSnord116*). The expression level of Snord116 observed in the *TgSnord116* mouse brain was lower than in wild type animals, but comparable to that in *PWScr*^*p*−/*m5*′*LoxP*^ mice (Supplementary Fig. 4). However, in contrast to *PWScr*^*p*−/*m5*′*LoxP*^, the *PWScr*^*p*−/*m*+^ *TgSnord116* mice once more failed to rescue the growth retardation phenotype. *In situ* hybridization experiments with a *Snord116 -* host specific probe revealed that *EGFP* expression was absent in thalamus, hypothalamus, midbrain and pons of *PWScr*^*p*−/*m*+^*TgSnord116* mice (brain areas where *Snord116* is expressed in wild-type; Supplementary Fig. 5). Dysregulation of the hypothalamic endocrine system has been shown to be associated with PWS in humans and could lead to growth retardation in mice^30^,^31^. Although, there is a possibility that the host transcript and Snord116 exhibit different stabilities in the aforementioned brain areas, it is conceivable that the absence of Snord116 in the hypothalamus of *PWScr*^*p*−/*m*+^ *TgSnord116* mice explains the lack of compensation for the PWS-like phenotype in these animals.

Thus, the important question of the functional significance of Snord116 C/D box snoRNAs in PWS still needs to be addressed by generating compensatory transgenic animals that express Snord116 in the same brain areas as do wild-type or *PWScr*^*p*−/*m5*′*LoxP*^ mice. In addition, based on recent findings, the impact of repetitive IPW-A exons containing non-protein coding RNA must be seriously considered.

The present study clearly demonstrates that the lack of expression of non-protein-coding RNAs from the PWS critical region is primarily causative of the growth retardation phenotype in mice. Importantly, the growth retardation observed in *PWScr*^*p*−/*m*+^ animals could be rescued by the transcriptional activation of the *PWScr* region from the silenced maternal chromosome. Our results suggest that activation of disease-associated genes on imprinted regions could lead to general therapeutic strategies in man. In fact, recent findings that topoisomerase inhibitors, such as topotecan, could activate the silent paternal Ube3a allele in neurons support this notion^32^,^33^.

## Material and Methods

### Generation of transgenic mice

Details on the 5′-LoxP targeting cassette construction were previously given^12^. In brief, two mouse genomic fragments generated from the *Snord116* upstream region were PCR amplified from a PAC clone (RPCIP711K19517Q6, RZPD German Resource Centre for Genome Investigation). These fragments were used as homologues arms in the targeting vector flanking the region containing the 5′-portion of the hypoxanthine-guanine phosphoribosyltransferase *(HPRT)* gene, the LoxP site and the neomycin resistance gene (Fig. 1B and Supplementary Fig. 1C)^12^. The resulting DNA construct was linearized with *Not*I endonuclease and electroporated at 25 μF and 400 V (gene Pulser; Bio-Rad) into AB2.2 embryonic stem (ES) cells (kindly provided by A. Bradley) resuspended in buffer containing 20 mM HEPES pH 7.4, 173 mM NaCl, 5 mM KCl, 0.7 mM Na_2_HPO_4_, 6 mM dextrose, and 0.1 mM ß-mercaptoethanol. ES cells, passage 17, were grown in HEPES-buffered, Dulbecco’s modified Eagle’s medium supplemented with 15% fetal bovine serum (HyClone), nonessential amino acids, L-glutamine, ß-mercaptoethanol, 1000 U/ml recombinant LIF (Chemicon) and antibiotics (penicillin 100 U/ml and streptomycin 100 μg/ml) on a g-irradiated monolayer of primary fibroblast feeder cells^12^. Positive ES clones were selected using a nested PCR approach and Southern blot hybridization with a ^32^P-labled 5′HR probe^12^. One KI ES clone containing an inserted 5′LoxP cassette was injected into 3.5-day-old B6D2F1 (C57BL/6 × DBA) blastocysts, and the resulting embryos were transferred to CD-1 foster mice. Chimeras were identified by their agouti coat color.

### Southern blot analysis

Positively targeted ES cell clones or mouse tail biopsies were analyzed by Southern-blotting. Approximately 5 μg of genomic DNA was digested with *Eco*RI (or *Eco*RV), fractionated on 0.8% agarose gels, and transferred to GeneScreen nylon membranes (NEN DuPont). The membranes were hybridized with a ^32^P-labeled 1.7-kb probe containing sequences 5′ to the targeted homology and washed with (final concentrations) 0.5x SSPE (1 × SSPE is 0.18 M NaCl, 10 mM NaH_2_PO4, and 1 mM EDTA [pH 7.7]) and 0.5% sodium dodecyl sulfate at 65 °C.

### Northern blot hybridization

Five μg of total RNA samples isolated from different organs of *PWScr*^*p*−/m5′LoxP^, *PWScrp−/m+* and wild type mice were separated on 8% (w/v) (acrylamid/N,N′-bis-acrylamide (29:1)) denaturing polyacrylamide gels (PAAG; 7 M urea, 1 × TBE buffer) and transferred onto positively charged nylon membranes (BrightStar Plus, Ambion or Hybond-N+, Amersham Biosciences) using a Trans-blot semi-dry blotting apparatus (BioRad) at 400 mA for 45 min in 0.5 × TBE buffer (90 mM Tris, 64.6 mM boric acid, 2.5 mM EDTA, pH 8.3). Membranes were first pre-hybridized for 30 min in 0.5 M sodium phosphate (pH 6.5), 7% (w/v) sodium dodecyl sulfate (SDS) buffer at 56 °C followed by overnight hybridization at the same temperature. Northern blot hybridization was performed with 5′-^32^P-labelled snoRNA specific oligonucleotides that were also used for reverse transcription in RT-qPCR assays (see below) (Supplementary Table 3). Membranes were washed twice at 46 °C in 2 × SSC buffer (20 mM sodium phosphate, 0.3 M NaCl, 2 mM EDTA, pH 7.4) containing 0.5% SDS for 30 min (washing was repeated when the blot showed high counts). Blots were exposed to MS-film (Kodak) for a few hours at −80 °C, if necessary, exposure time was extended overnight at −80 °C.

### RT qPCR analysis

Reverse transcription quantitative real-time PCR analysis was performed as previously reported^19^,^20^. Briefly, total RNA was isolated from mouse brains using TRIzol reagent (Invitrogen) according to the manufacturer’s instructions. RNA samples, 5 μg each, were treated with RNase-free DNase I (Roche), followed by reverse transcription with 0.5 μl of oligo(dT)_12–18_ (500 ng/μl) and 1 μl of random hexamer primer (3 μg/μl) oligonucleotide mix. All qPCR reactions were performed in triplicate, in a total volume of 10 μl containing 2 μl of cDNA (~20 ng), 5 μl of 2 × LightCycler 480 SYBR Green Master Mix (Roche) and 1 μM of each primer (Supplementary Table 3). The amplification program was as follows: 5 min initial denaturation step at 95 °C, with subsequent 45 cycles of 20 sec at 95 °C and 1 min at 60 °C. The reaction included single acquisition of fluorescent signal at 60 °C for each cycle and continuous acquisition from 50 °C to 97 °C at the end of the 45 cycles for melt-curve analysis. Quantification Cycle (Cq) values were calculated using Light-cycler 480 SW 1.5 software (Roche) and all data were transferred to Excel files for subsequent analysis. Data analysis was performed using a geometric mean of ActB mRNA and U1 snRNA selected as reference genes. The fold change is represented as 2^−ΔΔCq^ (Supplementary Table 2). The Minimum Information for Publication of Quantitative Real-Time PCR Experiments (MIQE) guidelines were followed in all qPCR experiments^34^.

### PWS-IC qPCR methylation analysis

Genomic DNA was isolated from mouse brains using the proteinase K and phenol/chloroform extraction method. Five μg of each methyl-sensitive endonuclease digestion DNA samples were incubated with 20 units of *Sac*II (Roche) restriction enzyme overnight at 37 °C. Quantitative real-time PCR reactions were performed in triplicate in a total volume of 10 μl containing 2 μl of DNA (1:50 dilution of *Sac*II digested or untreated DNA samples), 5 μl of 2 × LightCycler 480 SYBR Green Master Mix (Roche) and 1 μM of each primer. The amplification program and data acquisition was carried out as described above. Data analysis was performed using Snord64 as reference control. Fold change is represented as 2^−ΔΔCq^ (Supplementary Table 4).

### *In situ* hybridization

Mice were transcardially perfused with 0.1 M phosphate buffered saline (PBS, pH 7.2), followed by freshly prepared 4% paraformaldehyde in PBS (PFA). The brains were removed and fixed overnight in 4% PFA^35^. *In situ* hybridization was performed on floating 30 μm brain sections as previously described^36^. The Snord116 probe was synthesized *in vitro* and cloned into the pUC minusMCS plasmid, custom made by Blue Heron Biotech, LLC. The plasmid DNA was linearized with either *Bam*HI (for antisense probe) or *Sal*I (for sense probe) and transcribed *in vitro* in the presence of S^35^ α-UTP using T7 and T3 RNA polymerases, respectively. The ISH was performed at 50 °C overnight with the corresponding RNA probes ~4.5 × 10^6^ cpm/ml. The sections were washed once with 2X SSC and 50% deionized formamide buffer for 10 min at 50 °C followed by a single 10 min wash in 2X SSC at the same temperature and incubated with RNase A (90 ng/ml) for 45 min at 40 °C. Subsequently, the sections were washed by gentle stirring: first in 5 L of 2X SSC buffer for 45 min at 50 °C, then in 5 L of 0,1X SSC, 0,05% sodium pyrophosphate, 14 mM β-mercaptoethanol solution for 3 h at 50 °C, followed by a slow cooling of the buffer to room temperature and continued washing overnight. The slices were mounted on Superfrost Gold Plus (Menzel) microscopy glass slides, dried and exposed to autoradiography films (Kodak Biomax MR).

### Mice

All mouse procedures were performed in compliance with the guidelines for the welfare of experimental animals issued by the Federal Government of Germany and approved by the State Agency for Nature, Environment and Consumer Protection North Rhine-Westphalia (Landesamt für Natur, Umwelt und Verbraucherschutz Nordrhein-Westfalen). Animals were kept in specific pathogen-free animal facilities. All breeding was conducted in a controlled (21 °C, 30–50% humidity) room with a 12:12 hour light-dark cycle. Mice were housed under non-enriched, standard conditions in individually ventilated (36 (l) × 20 (w) × 20 (h) cm) cages for up to five littermates. Pups were weaned 19–23 days after birth and females were kept separately from males. Body weight statistic analysis was performed as previously described by Skryabin *et al.* 2007^12^.

## Additional Information

**How to cite this article**: Rozhdestvensky, T. S. *et al.* Maternal transcription of non-protein coding RNAs from the PWS-critical region rescues growth retardation in mice. *Sci. Rep.* **6**, 20398; doi: 10.1038/srep20398 (2016).

## Supplementary Material

Supplementary Information
