# Supplementary material for: Maternal transcription of non-protein coding RNAs from the PWS-critical region rescues growth retardation in mice
Source: Sci Rep. 2016 Feb 5;6:20398. doi: 10.1038/srep20398 (PMC4742849; doi:10.1038/srep20398)
Supplement: Supplementary Information [file srep20398-s1.pdf]

Supplementary information.

Maternal transcription of non-protein coding RNAs from the PWS-critical region rescues growth retardation in mice.

Timofey S. Rozhdestvensky[1,2*], Thomas Robeck[1,2], Chenna R. Galiveti[1], Carsten A. Raabe[1,3], Birte Seeger[1,2], Anna Wolters[1], Leonid V. Gubar[1,2], Jürgen Brosius[1,3] and Boris V. Skryabin[1,2*].

[1]Institute of Experimental Pathology (ZMBE), University of Muenster, Von-Esmarch-Str. 56, D-48149 Münster, Germany.

[2]Department of Medicine (TRAM), University Hospital of Muenster, Von-Esmarch-Str. 56, D-48149 Münster, Germany.

[3]Institute of Evolutionary and Medical Genomics, Brandenburg Medical School (MHB), D-16816 Neuruppin, Germany

[*]Corresponding author: Timofey S. Rozhdestvensky: rozhdest@uni-muenster.de; correspondence also can be addressed to Boris V. Skryabin: skryabi@uni-muenster.de.

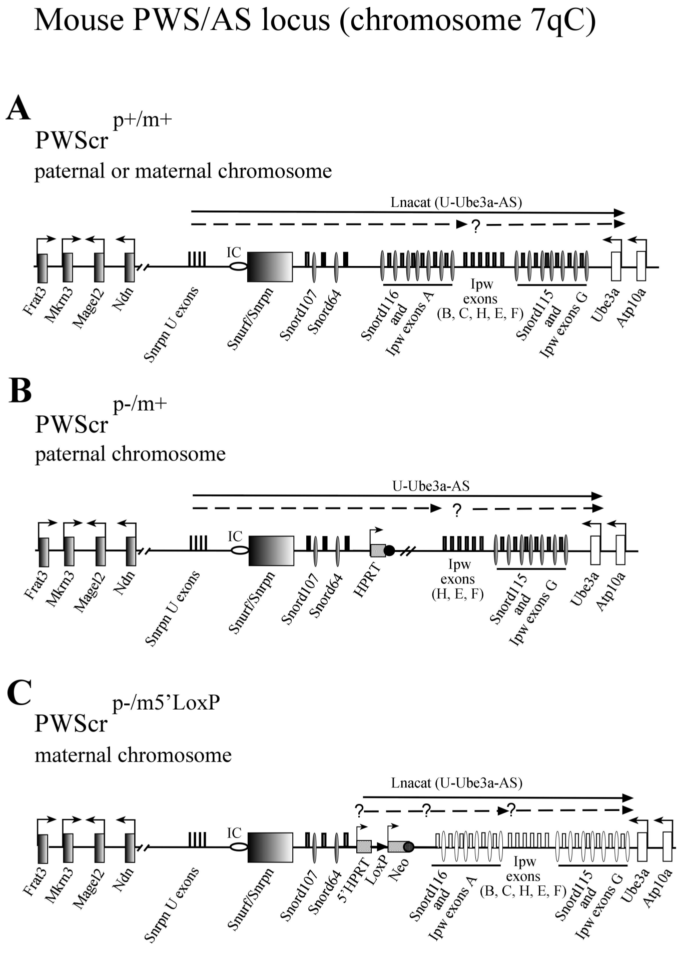

Supplementary Figure 1. Schematic representation of the mouse PWS/AS locus in investigated mice.

(**A**) Schematic representation of the PWS/AS locus on paternal or maternal chromosome in wild type mice. (**B**) Schematic representation of the PWS/AS locus on the paternal chromosome in *PWScr*$^{p-/m+}$ mice. (**C**) Schematic representation of the PWS/AS locus on maternal chromosome *in PWScr*$^{p-/m5'LoxP}$ mice. The PWS/AS locus on the paternal chromosome for this genotype is like on panel **B**. Drawing is not to scale. (**A**, **B**, **C**) Protein coding genes, snoRNAs and non-protein coding U-UBE3A-ATS exons are marked as boxes, ovals and bars, respectively. Paternally and maternally expressed genes in the PWS/AS locus are labelled black-grey and white, respectively. Known and predicted U-UBE3-ATS transcripts are shown as black and dashed arrows, respectively. Question marks depict regions with possible internal initiation of transcription. The PWS imprinting centre (PWS-IC) is schematically indicated by a white oval and marked with IC.

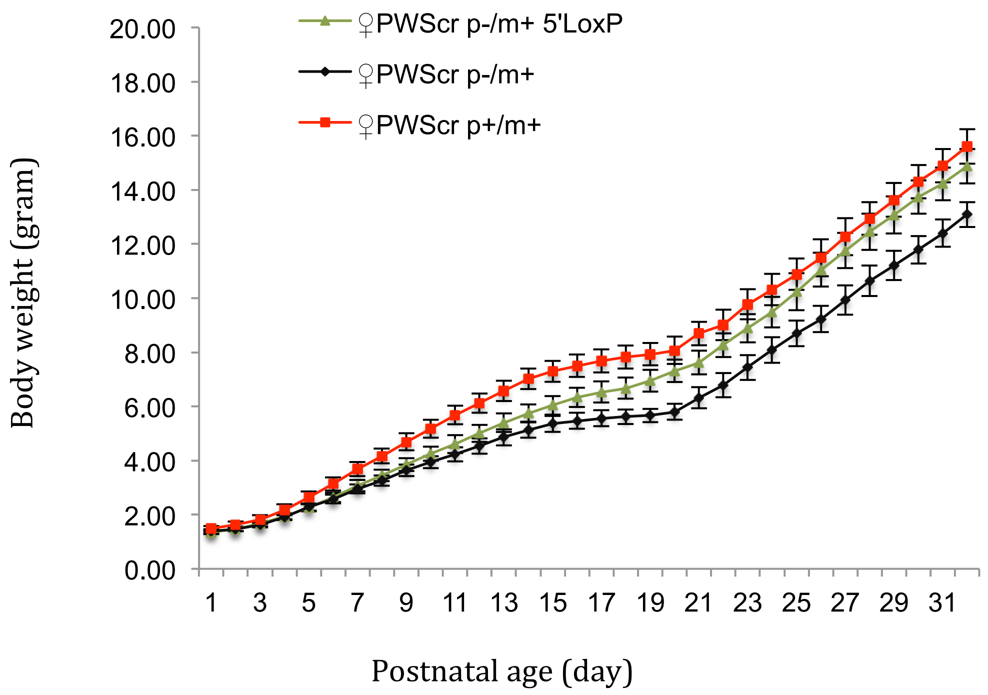

Supplementary Figure 2. Growth dynamics of PWScr$^{p-/m5'LoxP}$, PWScr$^{p-/m+}$ and wild type female mice.

The curves show the growth dynamics of 84 female mice. The red line corresponds to weight gain of 30 wild type mice. The yellow line corresponds to weight gain of 26 *PWScr*$^{p-/m5'LoxP}$ mice. The black line corresponds to weight gain of 28 *PWScr*$^{p-/m+}$ mice. Bars indicate standard deviation. Weights mean, observed standard deviation and confidence interval for each time point of investigated mice with the level of 95% are calculated in Supplementary Table 1.

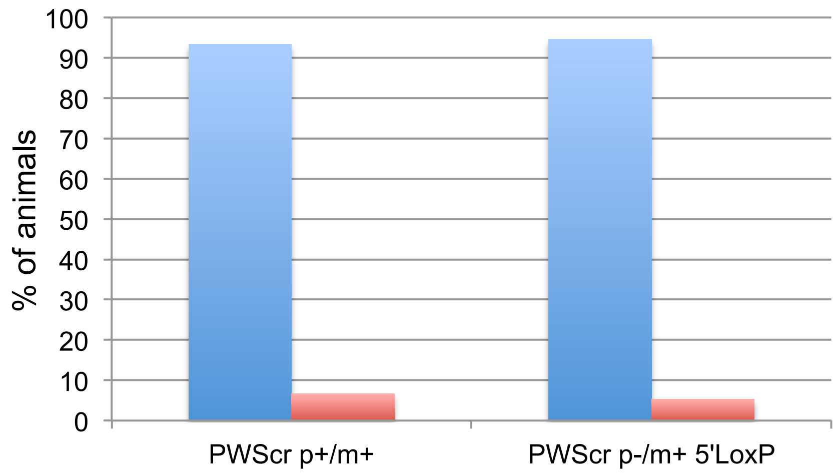

Supplementary Figure 3. Postnatal lethality of wild type *PWScr* $^{p+/m+}$ (n=60) and *PWScr* $^{p-/m+\ 5'LoxP}$ (n=56) mice. Blue and red bars represent percentages of surviving and dead mice, respectively, after 60 days of observation.

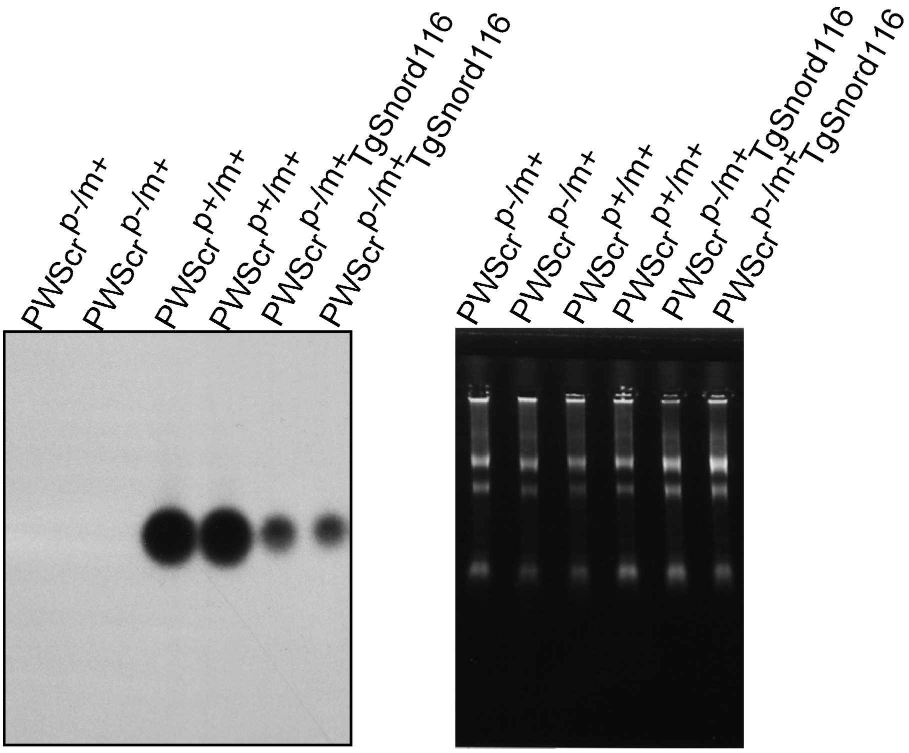

Supplementary Figure 4. Northern blot analyses of Snord116 in wild type, *PWScr*$^{p\text{-}/m+}$ and *PWScr*$^{p\text{-}/m+}$TgSnord116 mice (left panel). Total RNA isolated from mouse brains was separated on a 1.2% denaturing agarose gel. An ethidium bromide-stained gel is shown as RNA loading control (right panel). Mouse genotypes are indicated on the top of each lane.

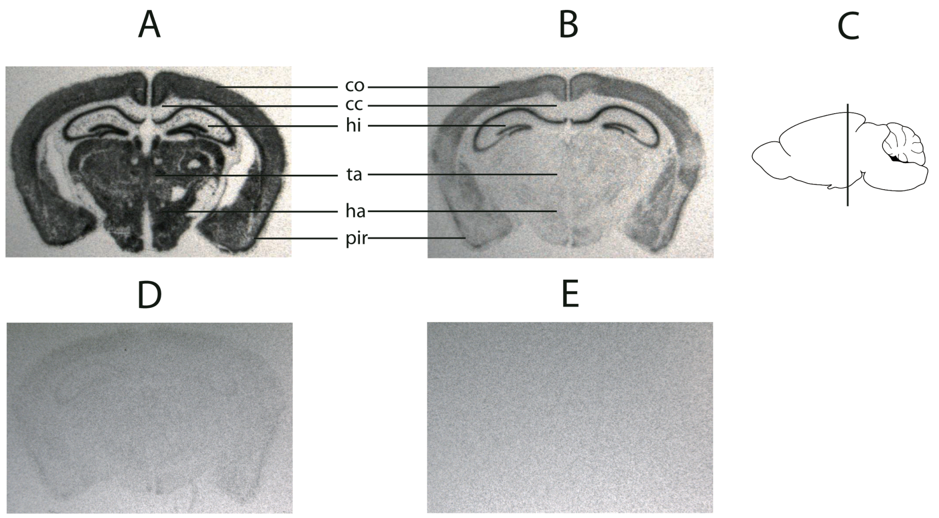

Supplementary Figure 5. *In situ* hybridization of wild type and *PWScr*$^{p-/m+}$TgSnord116 mouse sagittal brain sections.

(**A**) Brain sections of wild type mice. It is identical to the section shown on Figure 5A of the main manuscript. (**B**) Brain sections of *PWScr*$^{p-/m+}$TgSnord116 mice. (**A**, **B**) Mouse brain areas are denoted: cc: corpus callosum; co: cortex; ha: hypothalamic area; hi: hippocampus; ta: thalamic area; pir: piriformal cortex. (**C**) Schematic representation of the cutting in sagittal brain section. *In situ* hybridization was performed on floating 30µm brain sections as described in the main text. In case of *PWScr*$^{p-/m+}$TgSnord116 mice TgSnord116 specific probe complementary to the exons of transgenic Snord116 RNA's host transcript (eGFP) was used. (**D, E**) Examples of ISH control experiments. (**D**) Brain section of *PWScr*$^{p-/m+}$ TgSnord116 mice hybridized with eGFP sense probe. (**E**) Example of brain sections of wild type mice hybridized with eGFP antisense probe. (**B**, **D**, **E**) The exposure time was 4 days.

Supplementary Table 1. Growth dynamics of *PWScr*$^{p-/m5'LoxP}$, *PWScr*$^{p-/m+}$ and wild type mice.

Growth dynamics of *PWScr*$^{p-/m5'LoxP}$ male mice

Postnatal age (days)	*Weight mean (gram)*	*Standard deviation*	*Number of mice*	*A 95% Confidence interval*
1	1.43	0.16	27	0.06
2	1.56	0.19	28	0.07
3	1.75	0.23	30	0.08
4	2.02	0.27	30	0.10
5	2.40	0.35	30	0.13
6	2.78	0.35	30	0.13
7	3.19	0.42	30	0.15
8	3.62	0.45	30	0.16
9	4.06	0.49	30	0.18
10	4.45	0.57	30	0.20
11	4.92	0.72	30	0.26
12	5.22	0.70	30	0.25
13	5.59	0.73	30	0.26
14	5.95	0.76	30	0.27
15	6.23	0.79	30	0.28
16	6.52	0.81	30	0.29
17	6.70	0.89	30	0.32
18	6.89	0.89	30	0.32
19	7.18	0.92	30	0.33
20	7.63	0.87	30	0.31
21	8.11	0.88	30	0.31
22	8.60	1.00	30	0.36
23	9.38	0.93	30	0.33
24	10.18	1.10	30	0.39
25	10.98	1.10	30	0.39
26	11.82	1.26	30	0.45
27	12.80	1.39	30	0.50
28	13.73	1.45	30	0.52
29	14.73	1.48	30	0.53
30	15.75	1.67	30	0.60
31	16.63	1.65	30	0.59
32	17.66	1.71	30	0.61
33	18.50	1.72	30	0.62
34	19.10	1.81	30	0.65
35	19.68	1.77	30	0.63
36	19.93	1.73	30	0.62
37	20.16	1.89	30	0.68

38	20.69	1.78	30	0.64
39	21.11	1.85	30	0.66
40	21.40	1.86	30	0.67
41	21.56	1.83	30	0.65
42	22.10	1.89	30	0.68
43	22.27	1.75	30	0.63
44	22.41	1.96	30	0.70
45	22.82	1.80	30	0.64
46	23.17	1.87	30	0.67
47	23.33	1.77	30	0.63
48	23.70	1.81	30	0.65
49	23.81	1.77	26	0.68
50	24.05	1.81	26	0.70
51	24.18	1.81	26	0.70
52	24.45	1.81	26	0.70
53	24.53	1.81	26	0.70
54	24.70	1.89	26	0.73
55	24.93	2.05	26	0.79
56	24.97	2.12	26	0.82
57	25.14	1.87	26	0.72
58	25.34	2.06	26	0.79
59	25.54	2.01	26	0.77
60	25.69	1.92	26	0.74

Growth dynamics of *PWScr*$^{p-/m5'LoxP}$ female mice

**Postnatal age (days)**	***Weight mean (gram)***	***Standard deviation***	***Number of mice***	***A 95% Confidence interval***
1	1.35	0.17	26	0.07
2	1.47	0.21	26	0.08
3	1.67	0.25	26	0.10
4	1.94	0.30	26	0.11
5	2.26	0.38	26	0.14
6	2.64	0.43	26	0.17
7	3.07	0.53	26	0.21
8	3.45	0.55	26	0.21
9	3.85	0.62	26	0.24
10	4.25	0.67	26	0.26
11	4.61	0.87	26	0.34
12	5.00	0.80	26	0.31

13	5.40	0.88	26	0.34
14	5.74	0.86	26	0.33
15	6.04	0.92	26	0.35
16	6.34	0.95	26	0.36
17	6.53	1.01	26	0.39
18	6.67	1.00	26	0.39
19	6.94	1.04	26	0.40
20	7.31	1.07	26	0.41
21	7.62	1.14	26	0.44
22	8.26	1.14	26	0.44
23	8.89	1.37	26	0.53
24	9.48	1.46	26	0.56
25	10.24	1.76	26	0.68
26	11.05	1.61	26	0.62
27	11.75	1.70	26	0.65
28	12.45	1.76	26	0.67
29	13.08	1.78	26	0.69
30	13.74	1.62	26	0.62
31	14.22	1.60	26	0.61
32	14.87	1.65	26	0.63

Growth dynamics of *PWScr*$^{p-/m+}$ male mice

Postnatal age (days)	***Weight mean (gram)***	***Standard deviation***	***Number of mice***	***A 95% Confidence interval***
1	1.46	0.16	22	0.06
2	1.55	0.17	22	0.07
3	1.71	0.22	22	0.09
4	2.03	0.27	23	0.11
5	2.40	0.30	23	0.12
6	2.77	0.36	23	0.15
7	3.16	0.39	23	0.16
8	3.52	0.44	23	0.18
9	3.88	0.47	23	0.19
10	4.15	0.51	23	0.21
11	4.47	0.52	23	0.21
12	4.76	0.57	23	0.23
13	5.03	0.63	23	0.26
14	5.40	0.72	23	0.29
15	5.57	0.79	23	0.32
16	5.67	0.90	23	0.37
17	5.80	0.90	23	0.37

18	5.84	0.88	23	0.36
19	5.94	0.79	23	0.32
20	6.04	0.84	23	0.34
21	6.60	1.06	23	0.43
22	7.24	1.15	23	0.47
23	8.14	1.06	22	0.44
24	8.79	1.20	22	0.50
25	9.54	1.12	22	0.47
26	10.20	1.04	19	0.47
27	11.28	1.13	19	0.51
28	12.06	0.98	18	0.45
29	12.79	1.11	18	0.51
30	13.92	0.94	18	0.43
31	14.77	1.04	18	0.48
32	15.51	0.85	16	0.42
33	16.63	0.71	16	0.35
34	17.36	0.64	16	0.31
35	17.70	0.58	16	0.28
36	18.17	0.64	16	0.32
37	18.81	0.59	16	0.29
38	19.06	0.56	16	0.27
39	19.36	0.55	16	0.27
40	19.56	0.60	16	0.29
41	19.80	0.58	16	0.28
42	19.96	0.68	16	0.33
43	20.31	0.69	16	0.34
44	20.43	0.69	16	0.34
45	20.75	0.76	16	0.37
46	20.97	0.82	16	0.40
47	21.07	0.80	16	0.39
48	21.37	0.97	16	0.47
49	21.52	0.97	16	0.48
50	21.66	0.85	16	0.42
51	21.91	0.84	16	0.41
52	22.26	1.03	16	0.50
53	22.38	0.93	16	0.45
54	22.47	1.02	16	0.50
55	22.75	0.98	16	0.48
56	22.78	1.02	16	0.50
57	23.07	1.02	16	0.50
58	23.30	1.07	16	0.53
59	23.34	1.07	16	0.52
60	23.30	1.16	16	0.57

Growth dynamics of $PWScr^{p-/m+}$ female mice

Postnatal age (days)	*Weight mean (gram)*	*Standard deviation*	*Number of mice*	*A 95% Confidence interval*
1	1.39	0.17	26	0.07
2	1.47	0.20	26	0.08
3	1.63	0.25	26	0.10
4	1.91	0.30	28	0.11
5	2.28	0.39	28	0.14
6	2.58	0.43	28	0.16
7	2.96	0.46	28	0.17
8	3.26	0.55	28	0.20
9	3.63	0.57	28	0.21
10	3.94	0.60	28	0.22
11	4.22	0.69	28	0.26
12	4.54	0.78	28	0.29
13	4.86	0.80	28	0.30
14	5.14	0.82	28	0.30
15	5.36	0.80	28	0.30
16	5.47	0.81	28	0.30
17	5.56	0.78	28	0.29
18	5.62	0.73	28	0.27
19	5.67	0.69	28	0.25
20	5.80	0.79	28	0.29
21	6.32	1.04	28	0.39
22	6.79	1.21	28	0.45
23	7.44	1.24	28	0.46
24	8.08	1.28	28	0.48
25	8.70	1.29	28	0.48
26	9.23	1.30	27	0.49
27	9.93	1.42	27	0.54
28	10.64	1.42	25	0.56
29	11.21	1.38	25	0.54
30	11.79	1.29	25	0.51
31	12.40	1.28	25	0.50
32	13.09	1.14	24	0.46

Growth dynamics of wild type (*PWScr*$^{p+/m+}$) male mice

Postnatal age (days)	*Weight mean (gram)*	*Standard deviation*	*Number of mice*	*A 95% Confidence interval*
1	1.43	0.13	24	0.05
2	1.55	0.15	24	0.06
3	1.77	0.21	24	0.08
4	2.14	0.26	28	0.10
5	2.56	0.30	28	0.11
6	3.03	0.36	28	0.13
7	3.54	0.39	28	0.14
8	4.20	1.03	28	0.38
9	4.64	0.68	28	0.25
10	5.04	0.53	28	0.20
11	5.47	0.58	28	0.22
12	5.94	0.61	28	0.22
13	6.36	0.61	28	0.22
14	6.79	0.65	28	0.24
15	7.13	0.67	28	0.25
16	7.41	0.64	28	0.24
17	7.53	0.60	28	0.22
18	7.69	0.56	28	0.21
19	7.85	0.56	28	0.21
20	8.14	0.67	28	0.25
21	8.88	0.72	28	0.27
22	9.32	0.88	28	0.33
23	10.07	0.94	28	0.35
24	10.92	0.99	28	0.37
25	11.66	1.11	28	0.41
26	12.49	1.16	27	0.44
27	13.47	1.10	27	0.41
28	14.35	1.34	23	0.55
29	15.19	1.29	23	0.53
30	16.34	1.30	23	0.53
31	16.93	1.09	23	0.44
32	18.03	1.07	22	0.45
33	18.99	0.96	22	0.40
34	19.60	0.99	22	0.41
35	19.83	1.08	22	0.45
36	20.42	1.15	22	0.48
37	20.83	1.07	22	0.45
38	20.94	1.06	22	0.44
39	21.28	0.89	22	0.37
40	21.36	0.93	22	0.39

41	21.62	1.01	22	0.42
42	21.99	1.07	22	0.45
43	22.22	1.00	22	0.42
44	22.53	0.95	22	0.40
45	22.73	1.10	22	0.46
46	22.86	1.01	22	0.42
47	23.18	1.03	22	0.43
48	23.33	1.09	22	0.46
49	23.49	1.09	22	0.45
50	23.76	1.15	22	0.48
51	23.93	1.08	22	0.45
52	24.11	1.09	22	0.46
53	24.41	1.16	22	0.49
54	24.38	1.21	22	0.51
55	24.36	1.41	22	0.59
56	24.80	1.13	22	0.47
57	24.94	1.22	22	0.51
58	25.27	1.26	22	0.53
59	25.25	1.21	22	0.51
60	25.25	1.19	22	0.50

Growth dynamics of wild type (*$PWScr^{p+/m+}$*) female mice

**Postnatal age (days)**	***Weight mean (gram)***	***Standard deviation***	***Number of mice***	***A 95% Confidence interval***
1	1.49	0.25	30	0.09
2	1.62	0.34	30	0.12
3	1.82	0.44	30	0.16
4	2.18	0.56	30	0.20
5	2.64	0.63	30	0.23
6	3.14	0.69	30	0.25
7	3.68	0.72	30	0.26
8	4.17	0.79	30	0.28
9	4.69	0.88	30	0.31
10	5.17	0.91	30	0.33
11	5.68	0.94	30	0.34
12	6.12	1.02	30	0.36
13	6.57	1.03	30	0.37
14	7.02	1.05	30	0.38
15	7.30	1.10	30	0.39

16	7.50	1.16	30	0.42
17	7.68	1.20	30	0.43
18	7.83	1.20	30	0.43
19	7.93	1.15	30	0.41
20	8.07	1.39	30	0.50
21	8.69	1.21	30	0.43
22	9.01	1.56	30	0.56
23	9.77	1.58	30	0.56
24	10.31	1.63	30	0.58
25	10.88	1.61	30	0.58
26	11.49	1.73	25	0.68
27	12.27	1.72	25	0.68
28	12.94	1.57	25	0.61
29	13.63	1.57	25	0.62
30	14.30	1.55	25	0.61
31	14.89	1.58	25	0.62
32	15.61	1.56	23	0.64

Supplementary Table 2. RT-qPCR analyses of genes expression from the PWS-locus

Transcript Name	Genotype	Ave Cq (test gene)	StDev (test gene)	Ave Cq (control)	StDev (control)	ΔCq	StDev	ΔΔCq	$2^{-\Delta\Delta Cq}$	$2^{-\Delta\Delta Cq}$ +StDev	$2^{-\Delta\Delta Cq}$ -StDev
Frat3	WT	28.61	0.06	21.66	0.02	6.95	0.03	0.00	1.00	1.03	0.97
	KO	28.82	0.08	21.81	0.03	7.01	0.04	-0.06	1.04	1.08	1.00
	LoxP	29.28	0.05	22.07	0.03	7.21	0.03	-0.26	1.19	1.22	1.17
Mkrn3	WT	26.88	0.05	21.66	0.02	5.22	0.03	0.00	1.00	1.03	0.97
	KO	26.85	0.05	21.81	0.03	5.04	0.03	0.18	0.88	0.91	0.85
	LoxP	27.41	0.07	22.07	0.03	5.34	0.04	-0.11	1.08	1.12	1.05
Magel2	WT	26.96	0.07	21.66	0.02	5.31	0.04	0.00	1.00	1.04	0.96
	KO	27.24	0.06	21.81	0.03	5.43	0.03	-0.12	1.09	1.12	1.06
	LoxP	27.82	0.05	22.07	0.03	5.75	0.03	-0.44	1.36	1.39	1.33
Ndn	WT	20.54	0.01	21.66	0.02	-1.12	0.01	0.00	1.00	1.01	0.99
	KO	20.74	0.03	21.81	0.03	-1.07	0.02	-0.05	1.03	1.06	1.01
	LoxP	21.06	0.02	22.07	0.03	-1.01	0.01	-0.11	1.08	1.09	1.06
Snrpn_exon_U1	WT	23.74	0.03	21.66	0.02	2.09	0.02	0.00	1.00	1.02	0.98
	KO	23.93	0.03	21.81	0.03	2.12	0.02	-0.03	1.02	1.04	1.00
	LoxP	24.24	0.04	22.07	0.03	2.16	0.03	-0.07	1.05	1.08	1.03
Snrpn/snurf	WT	19.23	0.04	21.66	0.02	-2.43	0.02	0.00	1.00	1.02	0.98
	KO	19.34	0.01	21.81	0.03	-2.47	0.02	0.04	0.97	0.99	0.96
	LoxP	19.55	0.06	22.07	0.03	-2.53	0.03	0.10	0.93	0.97	0.90
Snurf assay	WT	21.37	0.04	21.66	0.02	-0.29	0.02	0.00	1.00	1.02	0.98
	KO	21.50	0.01	21.81	0.03	-0.31	0.01	0.02	0.99	1.00	0.97
	LoxP	21.68	0.03	22.07	0.03	-0.40	0.02	0.11	0.93	0.94	0.91
Snord64	WT	24.17	0.05	21.66	0.02	2.51	0.03	0.00	1.00	1.03	0.97
	KO	24.72	0.04	21.81	0.03	2.91	0.03	-0.39	1.31	1.34	1.29
	LoxP	24.94	0.04	22.07	0.03	2.86	0.02	-0.35	1.27	1.30	1.25

Transcript Name	Genotype	Ave Cq (test gene)	StDev (test gene)	Ave Cq (control)	StDev (control)	ΔCq	StDev	ΔΔCq	$2^{-\Delta\Delta Cq}$	$2^{-\Delta\Delta Cq}$ +StDev	$2^{-\Delta\Delta Cq}$ -StDev
IPW	WT	26.77	0.09	21.66	0.02	5.11	0.05	0.00	1.00	1.05	0.95
	KO	-	-	-	-	-	-	-	-	-	-
	LoxP	30.63	0.12	22.07	0.03	8.56	0.06	-3.44	10.89	10.95	10.83
IPW_exon_A1	WT	24.32	0.10	21.66	0.02	2.66	0.05	0.00	1.00	1.05	0.95
	KO	0.00	0.00	0.00	0.00	0.00	0.00	0.00	0.00	0.00	0.00
	LoxP	28.15	0.17	22.07	0.03	6.07	0.09	-3.41	10.63	10.72	10.54
Snord116	WT	21.43	0.02	21.66	0.02	-0.23	0.02	0.00	1.00	1.02	0.98
	KO	-	-	-	-	-	-	-	-	-	-
	LoxP	24.77	0.02	22.07	0.03	2.70	0.01	-2.92	7.59	7.61	7.58
IPW_exon_A2	WT	22.81	0.07	21.66	0.02	1.16	0.04	0.00	1.00	1.04	0.96
	KO	0.00	0.00	0.00	0.00	0.00	0.00	0.00	0.00	0.00	0.00
	LoxP	25.90	0.14	22.07	0.03	3.83	0.07	-2.67	6.38	6.46	6.31
IPW_exon_B	WT	26.26	0.05	21.66	0.02	4.60	0.03	0.00	1.00	1.03	0.97
	KO	-	-	-	-	-	-	-	-	-	-
	LoxP	30.28	0.21	22.07	0.03	8.20	0.11	-3.60	12.11	12.21	12.00
IPW_exon_F	WT	29.47	0.10	21.66	0.02	7.81	0.05	0.00	1.00	1.05	0.95
	KO	33.24	0.35	21.81	0.03	11.43	0.18	-3.62	12.30	12.48	12.12
	LoxP	33.33	0.41	22.07	0.03	11.26	0.20	-3.45	10.90	11.11	10.70
IPW_exon_G	WT	23.20	0.05	21.66	0.02	1.55	0.03	0.00	1.00	1.03	0.97
	KO	25.30	0.09	21.81	0.03	3.49	0.05	-1.94	3.84	3.89	3.80
	LoxP	25.63	0.08	22.07	0.03	3.56	0.04	-2.01	4.02	4.06	3.98
Snord115	WT	20.96	0.02	21.66	0.02	-0.70	0.01	0.00	1.00	1.01	0.99
	KO	22.11	0.01	21.81	0.03	0.30	0.02	-1.00	2.00	2.01	1.98
	LoxP	21.97	0.04	22.07	0.03	-0.10	0.03	-0.59	1.51	1.53	1.48

Transcript Name	Genotype	Ave Cq (test gene)	StDev (test gene)	Ave Cq (control)	StDev (control)	ΔCq	StDev	ΔΔCq	$2^{-ΔΔCq}$	$2^{-ΔΔCq}$ +StDev	$2^{-ΔΔCq}$ -StDev
Ube3a_AS_b	WT	29.06	0.12	21.66	0.02	7.40	0.06	0.00	1.00	1.06	0.94
	KO	29.59	0.12	21.81	0.03	7.78	0.06	-0.38	1.30	1.36	1.24
	LoxP	30.32	0.14	22.07	0.03	8.25	0.07	-0.84	1.79	1.86	1.72
Ube3a_AS_a	WT	29.75	0.08	21.66	0.02	8.09	0.04	0.00	1.00	1.04	0.96
	KO	30.65	0.17	21.81	0.03	8.84	0.09	-0.75	1.68	1.77	1.59
	LoxP	31.41	0.11	22.07	0.03	9.33	0.06	-1.24	2.36	2.42	2.30
Ube3a_long	WT	24.00	0.02	21.66	0.02	2.34	0.02	0.00	1.00	1.02	0.98
	KO	23.66	0.05	21.81	0.03	1.85	0.03	0.50	0.71	0.74	0.68
	LoxP	23.83	0.04	22.07	0.03	1.75	0.02	0.59	0.66	0.69	0.64
Ube3a_all	WT	23.70	0.08	21.66	0.02	2.05	0.04	0.00	1.00	1.04	0.96
	KO	23.47	0.06	21.81	0.03	1.66	0.03	0.39	0.76	0.80	0.73
	LoxP	23.53	0.01	22.07	0.03	1.46	0.01	0.59	0.66	0.68	0.65
Ube3a_13	WT	25.06	0.05	21.66	0.02	3.40	0.03	0.00	1.00	1.03	0.97
	KO	25.02	0.03	21.81	0.03	3.21	0.02	0.20	0.87	0.89	0.85
	LoxP	25.08	0.07	22.07	0.03	3.00	0.04	0.40	0.76	0.79	0.72
Atp10a	WT	30.68	0.15	21.66	0.02	9.02	0.08	0.00	1.00	1.08	0.92
	KO	30.58	0.10	21.81	0.03	8.77	0.05	0.25	0.84	0.89	0.79
	LoxP	31.37	0.29	22.07	0.03	9.30	0.14	-0.28	1.21	1.36	1.07

Supplementary Table 3: Details of oligonucleotides and hybridization probes used in this study

Used symbol	Name	Accession no.	**Primer sequences for RT-qPCR**	amplicon size (bp)
Frat3 (Peg12)	Paternally expressed 12		Forward: GTGACTGCATTCCACCTTTTC<br>Reverse: AGCGTCACCCCACTTGAGTA	60
Mkrn3	Makorin ring finger protein 3		Forward: GCCATTGCGGCAAGACTA<br>Reverse: TTCTTCTCAAGTGTAAGCGATCC	61
Magel 2	Melanoma antigen, family L, 2		Forward: CTCCAAGGCACTGCAAACT<br>Reverse: GCTGCACTGAAGGCAGTTCT	80
Ndn	Necdin		Forward: CAGAGAGGGTGCGGTCTG<br>Reverse: CTCCGAAGGTGGAGTGCTT	74
Snrpn_exon_1	Small nuclear Ribonucleoprtein N		Forward: TGCATTGGACAATATTCCTGTAG<br>Reverse: GCTCAGTGAGGCTGTCCTTC	123
Snurf assay	SNRPN upstream reading frame		Forward: GCCATTGCGGCAAGACTA<br>Reverse: TTCTTCTCAAGTGTAAGCGATCC	119
Snrpn/Snurf	Small nuclear Ribonucleoprtein N / SNRPN upstream reading frame		Forward: GGGCCCACCTCCTAAAGATA<br>Reverse: CCTCTGCCAGCTGCTCTT	60
Snord64	Small nuclear C/D box RNA 64		Forward: ACTTATGATGAGCTATGTTTACTG<br>Reverse: CTTCAGAGTAATCATTTTGAGC	62
IPW exons	IPW exons before snoRD116 cluster		Forward: TGCCCTAGGTCACTTGAGTTAAA<br>Reverse: GGGGTTTACTGGTAGCTCATTC	131
IPW_exon_A1	IPW exons between snoRD116 cluster		Forward: GTCCATGGAAAACATGCAGA<br>Reverse: ATGGAGACTCGGTTGGATGA	75
Snord116	Small nucleolar RNA, C/D box 116		Forward: TGATTCCCAGTCAAACATTCCT<br>Reverse: ACCTCAGTTCCGATGAGAGT	82
IPW_exon_A2	IPW exons between snoRD116 cluster		Forward: GCATTCCCTTTCCAGTATGGT<br>Reverse: TCGGTTGGACGACATAAACA	86
IPW_exon_B	IPW exons immediately after snoRD116 cluster		Forward: ACATCCAGAGAAAGTGTCACCA<br>Reverse: GGGTTTACTGGTAGCTCATTCAG	71
IPW_exon_F	IPW exons after snoRD116 cluster		Forward: CCATCTAAAATATTTCGACCCTGC<br>Reverse: GCTTCAGAGTTAGGAATGGTCAC	65
IPW_exon_G	IPW exons after snoRD116 cluster		Forward: GAAGACATTCTTCCAAGTATCAAG<br>Reverse: ATGGAACTGGAGAGGGATTG	84
Snord115	small nucleolar RNA, C/D box 115		Forward: GGTCAATGATGACAACCCAATG<br>Reverse: GCCTCAGCGTAATCCTATTGA	76
UBE3A-AS_b	UBE3A antisense region		Forward: TTGTATACAGGAAGCTAATGGGG<br>Reverse: CAAAAGTTTACAAATAAATAATGTTCC	157
UBE3A-_AS_a	UBE3A antisense region		Forward: ATCCATTCCACTAGTGTTTGCT<br>Reverse: GCCAACATGTAGCCTCTATATACTC	76
UBE3A_long	UBE3A		Forward: CTATACGAGGGAATCTGTTGTGATT<br>Reverse: CCTAGTCCTCCAACAGGTGCT	126
UBE3A_all	UBE3A		Forward: GAATCACTGTTCTTTACAGCCTAGTTC<br>Reverse: GGATTTTCCATAGCAATCATCTCT	127
UBE3A_1_3	UBE3A		Forward: AGGGCGACAAGGACCAGT<br>Reverse: TTCGGCTAGCTTCAATGTCC	113
ATP10a	Phospholipid-transporting ATPase VA		Forward: CGCCTGGAGACGAACTTG<br>Reverse: TTCCTGAAGGCGGTCTTCTAT	60

Used symbol	Name	Accession no.	**Primer sequences methylation qPCR analysis**	amplicon size (bp)
Snrpn	Small Nuclear RibonucleoProtein N	NM_033174	Forward: CCGCAGTGTCGCAGTCTGTT<br>Reverse: CTCATGCCCTGTAGCACATAGG	94
Snord64	C/D box Small Nucleolar RNA 64	NR_028529	Forward: ACTTATGATGAGCTATGTTTACTG<br>Reverse: CTTCAGAGTAATCATTTTGAGC	62

Used symbol	Name	**Probe sequences for Northern Blot analysis**
Snord116	C/D box Small nucleolar RNA 116 (MBII-85)	Reverse: ACCTCAGTTCCGATGAGAGT
Snord64	C/D box Small Nucleolar RNA 64 (MBII-13)	Reverse: CTTCAGAGTAATCATTTTGAGC
Snord115	C/D box small nucleolar RNA 115 (MBII-52)	Reverse: GCCTCAGCGTAATCCTATTGA

Used symbol	Name	**RNA Probe sequences for in situ hybridization**
Snord116 AS	MBII85 AS probe RNA	GGGUCGACCUCAGUUCCGAUGAGAGUGGCGGUACAG<br>AGUUUUCACUCAUUUUGUUCAGCUUUUCCAAGGAAU<br>GUUUGACUGGGAAUCAUCAUAGAUCG
Snord116 S	MBII85 sense probe RNA	GGGAUCCGAUCUAUGAUGAUUCCCAGUCAAACAUUC<br>CUUGGAAAAGCUGAACAAAAUGAGUGAAAACUCUGUA<br>CCGCCACUCUCAUCGGAACUGAGG
eGFP AS	eGFP AS probe RNA	GGGAACAAAAGCUGGGUACCGGGCCCCCCCUCGAGG<br>UCGACGGUAUCGAUAAGCUUGAUGGCGGCGGUCACG<br>AACUCCAGCAGGACCAUGUGAUCGCGCUUCUCGUUG<br>GGGUCUUUGCUCAGGGCGGACUGGGUGCUCAGGUA<br>GUGGUUGUCGGGCAGCAGCACGGGGCCGUCGCCGA<br>UGGGGGUGUUCUGCUGGUAGUGGUCGGCGAGCUGC<br>ACGCUGCCGUCCUCGAUGUUGUGGCGGAUCUUGAAG<br>UUCACCUUGAUGCCGUUCUUCUGCUUGUCGGCCAUG<br>AUAUAGACGUUGUGGCUGUUGUAGUUGUACUCCAGC<br>UUGUGCCCCAGGAUGUUGCCGUCCUCCUUGAAGUCG<br>AUGCCCUUCAGCUCGAUGCGGUUCACCAGGGUGUCG<br>CCCUCGAACUUCACCUCGGCGCGGGUCUUGUAGUUG<br>CCGUCGUCCUUGAAGAAGAUGGUGCGCUCCUGGACG<br>UAGCCUUCGGGCAUGGCGGACUUGAAGAAGUCGUGC<br>UGCUUCAUGUGGUCGGGGUAGCGGCUGAAGCACUGC<br>ACGCCGUAGGUCAGGGUGGUCACGAGGGUGGGCCA<br>GGGCACGGGCAGCUUGCCGGUGGUGCAGAUGAACUU<br>CAGGGUCAGCUUGCCGUAGGUGGCAUCGCCCUCGCC<br>CUCGCCGGACACGCUGAACUUGUGGCCGUUUACGUC<br>GCCGUCCAGCUCGACCAGGAUGGGCACCACCCCGGU<br>GAACAGCUCCUCGCCCUUGCUCACCAUGCUUCCCAC<br>UUUGCGUUUCUUCUUGGGCAUCGAAUUCCUGCAGCC<br>CGGGG
eGFP S	eGFP sense probe RNA	GGGAACAAAAGCUGGGUACCGGGCCCCCCCUCGAGG<br>UCGACGGUAUCGAUAAGCUUGAUGCCCAAGAAGAAAC<br>GCAAAGUGGGAAGCAUGGUGAGCAAGGGCGAGGAGC<br>UGUUCACCGGGGUGGUGCCCAUCCUGGUCGAGCUG<br>GACGGCGACGUAAACGGCCACAAGUUCAGCGUGUCC<br>GGCGAGGGCGAGGGCGAUGCCACCUACGGCAAGCUG<br>ACCCUGAAGUUCAUCUGCACCACCGGCAAGCUGCCC<br>GUGCCCUGGCCCACCCUCGUGACCACCCUGACCUAC<br>GGCGUGCAGUGCUUCAGCCGCUACCCCGACCACAUG

AAGCAGCACGACUUCUUCAAGUCCGCCAUGCCCGAA
GGCUACGUCCAGGAGCGCACCAUCUUCUUCAAGGAC
GACGGCAACUACAAGACCCGCGCCGAGGUGAAGUUC
GAGGGCGACACCCUGGUGAACCGCAUCGAGCUGAAG
GGCAUCGACUUCAAGGAGGACGGCAACAUCCUGGGG
CACAAGCUGGAGUACAACUACAACAGCCACAACGUCU
AUAUCAUGGCCGACAAGCAGAAGAACGGCAUCAAGGU
GAACUUCAAGAUCCGCCACAACAUCGAGGACGGCAG
CGUGCAGCUCGCCGACCACUACCAGCAGAACACCCC
CAUCGGCGACGGCCCCGUGCUGCUGCCCGACAACCA
CUACCUGAGCACCCAGUCCGCCCUGAGCAAAGACCC
CAACGAGAAGCGCGAUCACAUGGUCCUGCUGGAGUU
CGUGACCGCCGCCAUCGAAUUCCUGCAGCCCGGGG

Supplementary Table 4. qPCR results for PWS-IC-center CpG methylation analysis.

Sample Name	IC AVE Cq (tech. rep)	IC St. Dev (tech. rep)	Snord64 AVE Cq (tech. rep)	Snord64 St. Dev (tech. rep)	DCq	St. Dev
WT1_SacII	24.570	0.046	23.730	0.062	-0.840	0.039
WT2_SacII	24.073	0.023	23.140	0.036	-0.933	0.021
WT3_SacII	23.943	0.067	23.123	0.040	-0.820	0.039
WT4_SacII	23.893	0.023	23.033	0.006	-0.860	0.012
WT5_SacII	24.217	0.023	23.300	0.030	-0.917	0.019
WT6_SacII	23.827	0.006	22.917	0.023	-0.910	0.012
WT1_Uncut	23.580	0.050	23.683	0.042	0.103	0.033
WT2_Uncut	22.627	0.038	22.763	0.104	0.137	0.055
WT3_Uncut	22.997	0.021	23.107	0.047	0.110	0.026
WT4_Uncut	23.117	0.038	23.313	0.222	0.197	0.113
WT5_Uncut	23.113	0.032	23.170	0.017	0.057	0.018
WT6_Uncut	22.667	0.046	22.807	0.047	0.140	0.033
KO1_SacII	23.957	0.025	23.133	0.065	-0.823	0.035
KO2_SacII	24.383	0.067	23.573	0.035	-0.810	0.038
KO3_SacII	24.063	0.015	23.447	0.126	-0.617	0.063
KO4_SacII	23.567	0.055	22.690	0.010	-0.877	0.028
KO5_SacII	26.287	0.273	25.823	0.015	-0.463	0.137
KO6_SacII	23.087	0.057	22.270	0.053	-0.817	0.039

KO1_Uncut	22.950	0.044	22.993	0.105	0.043	0.057
KO2_Uncut	23.497	0.064	23.717	0.015	0.220	0.033
KO3_Uncut	23.207	0.029	23.537	0.021	0.330	0.018
KO4_Uncut	22.623	0.067	22.787	0.031	0.163	0.037
KO5_Uncut	25.610	0.046	25.947	0.035	0.337	0.029
KO6_Uncut	22.097	0.131	22.433	0.006	0.337	0.065
LoxP1_SacII	24.423	0.090	23.653	0.029	-0.770	0.047
LoxP2_SacII	24.257	0.038	23.500	0.046	-0.757	0.030
LoxP3_SacII	23.743	0.042	22.887	0.050	-0.857	0.033
LoxP4_SacII	24.117	0.080	23.310	0.044	-0.807	0.046
LoxP5_SacII	23.670	0.017	22.900	0.030	-0.770	0.017
LoxP6_SacII	23.660	0.046	22.810	0.010	-0.850	0.023
LoxP1_Uncut	23.793	0.055	23.983	0.025	0.190	0.030
LoxP2_Uncut	23.120	0.030	23.463	0.021	0.343	0.018
LoxP3_Uncut	22.863	0.031	22.947	0.035	0.083	0.023
LoxP4_Uncut	22.477	0.006	22.647	0.084	0.170	0.042
LoxP5_Uncut	22.237	0.025	22.560	0.026	0.323	0.018
LoxP6_Uncut	22.737	0.023	22.947	0.045	0.210	0.025
